# Clinical Relevance and Potential Applications of Brachial-Ankle Pulse Wave Velocity: Current Evidence and Future Perspectives

**DOI:** 10.3390/jcm15176689

**Published:** 2026-08-28

**Authors:** Hack-Lyoung Kim

**Affiliations:** Division of Cardiology, Department of Internal Medicine, Seoul Metropolitan Government-Seoul National University Boramae Medical Center, Seoul National University College of Medicine, 5 Boramae-ro, Dongjak-gu, Seoul 07061, Republic of Korea; khl2876@gmail.com or khl2876@snu.ac.kr

**Keywords:** arterial stiffness, brachial-ankle pulse wave velocity, cardiovascular risk, target organ damage, vascular aging

## Abstract

Arterial stiffness is increasingly recognized as an important marker of vascular aging and an independent predictor of cardiovascular disease. Among the available methods for assessing arterial stiffness, brachial-ankle pulse wave velocity (baPWV) has gained widespread clinical acceptance because it is simple, noninvasive, highly reproducible, and suitable for routine practice and large-scale population screening. Accumulating evidence has demonstrated that baPWV is associated with hypertension-mediated target organ damage, coronary artery disease, cardiovascular events, and mortality. Beyond its prognostic value, baPWV has been investigated as a potential marker of vascular responses to pharmacological and lifestyle interventions. Emerging evidence also suggests that serial assessment and visit-to-visit variability of baPWV may provide additional prognostic information, although their clinical applicability requires further validation. Another advantage of baPWV is its simultaneous measurement of the ankle-brachial index, enabling comprehensive assessment of both arterial stiffness and peripheral artery disease without additional examination time. This review summarizes the physiological basis, measurement principles, strengths and limitations, and current clinical applications of baPWV, with particular emphasis on cardiovascular risk stratification, target organ damage, therapeutic monitoring, serial assessment, and combined ankle-brachial index evaluation. Finally, future perspectives for integrating baPWV into precision cardiovascular medicine are discussed.

## 1. Introduction

Population aging has become a global phenomenon, accompanied by a substantial increase in the burden of cardiovascular disease (CVD) [1]. Although traditional cardiovascular risk factors such as hypertension, diabetes mellitus, dyslipidemia, and smoking remain important determinants of cardiovascular outcomes, growing evidence suggests that vascular aging itself represents a fundamental biological process underlying the development of CVD [2]. Among the manifestations of vascular aging, arterial stiffness has emerged as a key marker reflecting cumulative vascular damage from aging and long-term exposure to cardiovascular risk factors [3,4].

Over the past two decades, arterial stiffness assessment has evolved from a research tool to a clinically relevant method for cardiovascular risk evaluation. Carotid-femoral pulse wave velocity (cfPWV) is widely regarded as the reference standard for measuring arterial stiffness [5]. However, technical complexity, the need for specialized training, and relatively time-consuming procedures limit its use in routine clinical practice [6]. In contrast, brachial-ankle pulse wave velocity (baPWV) offers a simple, noninvasive, and highly reproducible alternative that can be measured rapidly using automated devices [6]. As a result, baPWV has gained widespread acceptance, particularly in East Asian countries, where extensive epidemiological and clinical studies have established its association with target organ damage, cardiovascular events, and mortality [7,8,9].

Given growing interest in vascular aging and the growing evidence supporting the prognostic value and potential clinical relevance of baPWV, a comprehensive review is timely. This review summarizes the physiological basis, measurement principles, strengths and limitations, and current clinical applications of baPWV, with a particular focus on its role in cardiovascular risk stratification and disease management.

## 2. Literature Search Strategy

This narrative review was based on a literature search of PubMed/MEDLINE and Web of Science for articles published from database inception through July 2026. The final literature search was conducted on 23 July 2026. Search terms were combined using Boolean operators (AND/OR) according to the specific topic of interest. A representative search strategy was: (“brachial-ankle pulse wave velocity” OR “baPWV”) AND (“arterial stiffness” OR “vascular aging”) AND (“cardiovascular disease” OR “cardiovascular events” OR “mortality”). Additional topic-specific terms, including “coronary artery disease,” “target organ damage,” “left ventricular hypertrophy,” “diastolic dysfunction,” “chronic kidney disease,” “cerebrovascular disease,” “cognitive impairment,” “hypertension,” “therapeutic response,” “serial measurement,” “variability,” and “ankle-brachial index,” were combined with the principal baPWV terms as appropriate. Reference lists of relevant reviews, meta-analyses, guidelines, consensus statements, and key original articles were also screened to identify additional pertinent studies.

The search was restricted to English-language articles. Given the narrative nature of this review, a formal systematic-review protocol was not applied. Studies were selected based on their relevance to the physiological basis, measurement characteristics, clinical associations, prognostic value, and potential clinical applications of baPWV. Priority was given to international guidelines and expert consensus statements, systematic reviews and meta-analyses, large prospective cohort studies, and well-designed original investigations. For topics with limited available evidence, smaller observational or mechanistic studies were also considered. Studies that were not directly relevant to baPWV or its clinical interpretation were excluded. When multiple studies addressed similar clinical questions, representative studies were selected based on methodological quality, sample size, prospective design, duration of follow-up, clinical relevance of the endpoints, and adequacy of multivariable adjustment. For studies evaluating cardiovascular prognosis, preference was also given to those reporting incremental predictive measures, when available.

## 3. Concept of Arterial Stiffness and Vascular Aging

Arterial stiffness reflects the progressive loss of the arterial wall’s elastic properties and has emerged as a central concept in the framework of vascular aging. The large elastic arteries normally buffer the heart’s pulsatile output, cushioning the pressure wave and ensuring steady perfusion of peripheral organs [10]. With advancing age, this cushioning function deteriorates as the wall’s structural composition changes [11,12]. The hallmark process is fragmentation and degradation of elastin fibers, driven by repetitive mechanical fatigue and matrix metalloproteinase activation, accompanied by collagen deposition and cross-linking. The accumulation of advanced glycation end-products further stiffens collagen, while medial calcification and chronic low-grade inflammation accelerate the transition toward a rigid conduit. Endothelial dysfunction compounds these structural changes by reducing nitric oxide bioavailability and increasing vascular tone, linking functional and structural components of stiffening [13,14]. Together, these processes embody what is increasingly described as “early vascular aging (EVA)”, a phenotype in which arterial stiffness exceeds that expected for chronological age and identifies individuals at heightened cardiovascular risk [15].

A crucial distinction exists between central elastic arteries, such as the aorta, and peripheral muscular arteries, such as the brachial and femoral vessels [10]. Central arteries depend primarily on elastin for their compliance and stiffen predominantly through age-related structural remodeling. Peripheral muscular arteries, by contrast, are strongly influenced by smooth muscle tone and are more sensitive to functional, neurohumoral, and metabolic regulation [3,16]. This regional heterogeneity is essential for interpreting any stiffness measurement, because different indices capture different vascular territories [16].

Beyond its role as a marker of vascular aging, arterial stiffness has important hemodynamic and clinical consequences. As arterial stiffness increases, pulse wave velocity accelerates and reflected waves return to the central aorta earlier during systole rather than diastole. This increases central systolic blood pressure, widens pulse pressure, and increases left ventricular afterload. At the same time, reduced diastolic pressure may impair coronary perfusion. These alterations contribute to left ventricular hypertrophy, myocardial ischemia, heart failure, and microvascular damage in vulnerable organs such as the brain and kidneys [6,17]. Consequently, arterial stiffness is increasingly recognized not merely as a consequence of aging but as an active mediator of cardiovascular disease development and progression.

## 4. The Measurement of Arterial Stiffness

Several methods have been developed to assess arterial stiffness. Among these, pulse wave velocity (PWV) is the most widely accepted and validated approach [18]. PWV is based on the principle that pulse waves travel more rapidly through stiff arteries than through compliant arteries. Therefore, the velocity of pulse wave propagation serves as a direct marker of arterial stiffness and vascular aging [6]. Carotid-femoral pulse wave velocity (cfPWV) is widely regarded as the reference standard for noninvasive assessment of arterial stiffness [5]. It measures the transit time of the pulse wave between the carotid and femoral arteries, thereby primarily reflecting the stiffness of the aorta and other central elastic arteries. Numerous epidemiological studies and meta-analyses have demonstrated that cfPWV independently predicts cardiovascular events, cardiovascular mortality, and all-cause mortality [19,20,21,22,23]. Consequently, both European and international expert consensus documents recommend cfPWV as the preferred measure of aortic stiffness [5,24].

Despite its strong evidence base, routine use of cfPWV in clinical practice remains limited. The procedure requires accurate assessment of carotid and femoral pulse waveforms, exposure of the inguinal region, and operator expertise [6]. These factors may reduce patient acceptance and limit widespread implementation, particularly in large-scale screening programs. To overcome these limitations, several alternative indices have been developed. Among them, baPWV has gained considerable popularity, especially in East Asian countries. baPWV is obtained by simultaneously recording pulse waveforms at the brachial arteries and ankles using automated oscillometric cuffs. The measurement is simple, rapid, highly reproducible, and requires minimal operator training [25,26]. Unlike cfPWV, which predominantly reflects central arterial stiffness, baPWV captures pulse wave transmission through both central elastic arteries and peripheral muscular arteries, providing a more global assessment of the arterial tree [27].

Another increasingly used index is the cardio-ankle vascular index (CAVI), which evaluates arterial stiffness over a broad arterial segment from the aortic origin to the ankle. CAVI combines PWV and blood pressure into a stiffness parameter designed to reduce dependence on blood pressure at the time of measurement, representing a potential advantage over baPWV, which is more directly influenced by concurrent blood pressure [28,29]. However, its prognostic evidence remains less extensive than that of cfPWV and baPWV. However, CAVI and baPWV are based on different physiological assumptions and algorithms and should not be considered interchangeable. Compared with CAVI, baPWV offers methodological simplicity, widespread use in East Asia, extensive epidemiological and prognostic evidence, and simultaneous ABI measurement, although direct comparative evidence demonstrating the superiority of either index for cardiovascular risk prediction remains limited. Other approaches for evaluating arterial stiffness include local measurements using ultrasound-based arterial distensibility, magnetic resonance imaging, and augmentation index derived from pulse wave analysis [18]. Although these techniques provide valuable physiological information, their complexity and limited availability may limit routine clinical use.

## 5. Standardized Measurement and Factors Affecting baPWV

Accurate, reproducible baPWV assessment requires standardized measurement conditions. Measurements should preferably be performed in a quiet, temperature-controlled room after at least 5–10 min of rest in the supine position. Avoid recent vigorous exercise, smoking, caffeine intake, alcohol consumption, and heavy meals before measurement because these factors may transiently alter vascular tone and hemodynamic conditions. Apply appropriately sized cuffs to both upper arms and ankles, paying close attention to cuff positioning, particularly in patients with obesity or large arm circumference. Because baPWV is substantially influenced by blood pressure and, to a lesser extent, heart rate at the time of measurement, record these parameters and consider them when interpreting between-individual or serial changes in baPWV [16,24,30].

baPWV is calculated by dividing the estimated arterial path length from the brachium to the ankle by the pulse transit time. In commonly used automated devices, arterial path lengths from the suprasternal notch to the brachium and ankle are estimated from the participant’s height using validated equations, while transit time is determined from the time delay between the initial upstrokes of the brachial and ankle pulse waveforms using a foot-to-foot method [24,31,32]. Although height-based estimation facilitates automated measurement, it may overestimate the actual arterial path length compared with magnetic resonance imaging-based measurements, which should be considered when comparing absolute baPWV values with other PWV indices [31].

Measurements are obtained bilaterally, and previous studies have used either the mean or the higher value of the right- and left-sided baPWV. Therefore, the method used to derive a representative baPWV value should be clearly specified and consistently applied [33,34].

Several clinical conditions may compromise baPWV validity or interpretation. Peripheral artery disease, particularly when the ankle-brachial index is <0.90, may delay pulse-wave transmission distal to an arterial stenosis and thereby artifactually lower baPWV. Arrhythmias, particularly atrial fibrillation or frequent ectopic beats, may increase beat-to-beat variability and reduce measurement reproducibility. Severe arterial calcification may interfere with arterial compressibility and pulse-wave detection, while marked obesity may compromise appropriate cuff fitting and waveform quality. Substantial inter-arm blood pressure differences may indicate subclavian or other upper-extremity arterial disease and should also prompt cautious interpretation of bilateral baPWV measurements [16,24,30,35]. These factors should therefore be considered when determining whether an individual baPWV measurement is technically reliable and clinically interpretable.

## 6. baPWV: Strengths and Limitations

baPWV offers several practical advantages that have facilitated its widespread clinical adoption. The measurement is fully automated, noninvasive, rapid, and highly reproducible, making it suitable for routine outpatient practice, health screening programs, and large epidemiological studies [6,7,8,9,25]. Despite its methodological simplicity, numerous studies across diverse populations have demonstrated its prognostic value for cardiovascular events and mortality [7,8,9]. In addition, baPWV allows simultaneous measurement of blood pressure and the ankle-brachial index (ABI), further enhancing its practical utility [25].

A distinctive feature of baPWV is that it reflects both central and peripheral arterial stiffness. Although muscular arteries were historically considered a limitation because they offer less specificity for central aortic stiffness [5], accumulating evidence suggests that peripheral muscular arterial remodeling is also associated with cardiovascular risk [36,37]. Thus, the broader vascular territory captured by baPWV may contribute to its prognostic value, although this should not be interpreted as evidence of superiority over measures of central arterial stiffness [8,27,38].

Several baPWV thresholds have been proposed for cardiovascular risk stratification. A value of approximately 1400 cm/s has been proposed to indicate increased arterial stiffness or early vascular damage, whereas values around 1800 cm/s have been associated with a higher burden of target-organ damage and cardiovascular risk [24]. These values provide a practical hierarchical framework in which progressively higher baPWV generally indicates greater vascular and cardiovascular risk. However, they should be regarded as proposed risk-stratification thresholds rather than universally validated diagnostic or treatment cutoffs. Importantly, these thresholds have been derived and validated predominantly in East Asian populations and should not be automatically extrapolated to other ethnic or geographic populations without external validation. Population-specific calibration may also be necessary before their broader clinical application. baPWV values are influenced by age, concurrent blood pressure, population characteristics, and measurement methodology. Moreover, researchers have not established robust, externally validated, disease-specific thresholds for coronary artery disease and cardiac, renal, or cerebral target-organ damage. Reported organ-specific cutoffs vary considerably across individual cohorts and therefore cannot currently be recommended for routine clinical use. Further prospective validation across diverse populations is required to establish clinically meaningful general and disease-specific thresholds.

Despite its advantages, baPWV has important limitations. Most notably, it is influenced by blood pressure at the time of measurement and therefore requires interpretation in the context of concurrent hemodynamic conditions [5]. Significant peripheral artery disease may artifactually lower baPWV by delaying pulse-wave transmission to the ankle, while arrhythmias, marked inter-arm blood pressure differences, and severe obesity may compromise measurement reliability. Furthermore, because baPWV incorporates both central and peripheral arterial segments, it should not be interpreted as a direct measure of aortic stiffness [5,39].

The principal differences between cfPWV and baPWV are summarized in Table 1. cfPWV directly assesses the carotid-to-femoral arterial segment and remains the reference-standard noninvasive measure of central aortic stiffness, whereas baPWV provides a broader assessment of central and peripheral arterial stiffness using automated four-limb oscillometric measurements. Thus, the methodological convenience of baPWV should not be interpreted as physiological equivalence to cfPWV, as the two measures represent different arterial territories and aspects of vascular stiffness.

Accordingly, cfPWV is preferable when direct assessment of central aortic stiffness is the primary objective, whereas baPWV’s simplicity, scalability, and simultaneous ABI measurement make it particularly suitable for routine clinical assessment, screening programs, and epidemiological studies. Both measures have demonstrated prognostic associations with cardiovascular morbidity and mortality; however, cfPWV has been validated across more diverse populations and remains the arterial stiffness measure most widely recognized in international guidelines, whereas much of the evidence for baPWV originates from East Asian populations. Although baPWV has provided incremental prognostic information beyond conventional risk factors in several cohorts, direct head-to-head comparisons using identical populations, endpoints, and statistical models remain limited. Therefore, current evidence does not establish consistent prognostic superiority of either measure across specific cardiovascular diseases.

## 7. baPWV as a Marker of Target Organ Damage

### 7.1. Coronary Artery Disease

Coronary artery disease (CAD) is one of the most clinically important manifestations of target organ damage resulting from long-standing arterial hypertension and vascular aging. Increased arterial stiffness raises systolic blood pressure and lowers diastolic blood pressure. The increased systolic blood pressure augments left ventricular afterload, promoting left ventricular hypertrophy and increasing myocardial oxygen demand, which may result in subendocardial ischemia. Conversely, reduced diastolic blood pressure compromises coronary perfusion, as coronary blood flow occurs predominantly during diastole, contributing to coronary underperfusion. In addition, arterial stiffness shares many traditional cardiovascular risk factors, including aging, hypertension, diabetes mellitus, dyslipidemia, and smoking, all of which contribute to the development and progression of coronary atherosclerosis [6,40]. Beyond reflecting cumulative vascular damage, increased arterial stiffness is also believed to play a direct pathogenic role in atherosclerosis by increasing pulsatile mechanical stress on the arterial wall and promoting endothelial dysfunction, vascular inflammation, and adverse vascular remodeling. Increased arterial stiffness is associated with a greater atherosclerotic burden and may expose the arterial wall and atherosclerotic plaques to increased pulsatile and mechanical stress [41]. Although these hemodynamic alterations may contribute to plaque instability, direct evidence establishing arterial stiffness as a causal determinant of plaque rupture remains limited. Several observational studies have demonstrated that higher baPWV is independently associated with the presence and severity of CAD, even after adjustment for traditional cardiovascular risk factors [42,43,44,45]. Furthermore, baPWV may provide incremental diagnostic information when noninvasive imaging studies are inconclusive or have limited ability to predict CAD, thereby improving CAD diagnostic accuracy [46].

### 7.2. Left Ventricular Hypertrophy

baPWV is closely associated with left ventricular hypertrophy (LVH), a well-established marker of hypertensive target organ damage and an independent predictor of cardiovascular morbidity and mortality [47,48]. Increased arterial stiffness, reflected by elevated baPWV, augments systolic blood pressure and pulse pressure, thereby increasing left ventricular afterload. This chronic hemodynamic burden promotes compensatory myocardial hypertrophy and concentric left ventricular remodeling [6,17]. Numerous cross-sectional and longitudinal studies have demonstrated that elevated baPWV is independently associated with increased left ventricular mass index (LVMI) and the presence of LVH after adjustment for conventional cardiovascular risk factors, including age and blood pressure [49,50,51,52,53]. Importantly, the association between baPWV and LVH extends beyond the effect of blood pressure alone, suggesting that arterial stiffness contributes directly to myocardial structural remodeling through both hemodynamic and vascular-biological mechanisms. However, these observational associations do not establish a direct causal relationship between arterial stiffness and myocardial hypertrophy. Collectively, these findings support baPWV as a vascular marker associated with hypertensive cardiac remodeling and highlight the close relationship between vascular stiffening and myocardial structural alterations.

### 7.3. Left Ventricular Diastolic Dysfunction

Left ventricular diastolic dysfunction is one of the earliest manifestations of hypertension-mediated cardiac target organ damage and often precedes the development of heart failure with preserved ejection fraction [54,55,56,57]. Increased arterial stiffness, as reflected by elevated baPWV, increases central systolic blood pressure, pulse pressure, and left ventricular afterload, thereby promoting left ventricular hypertrophy and concentric remodeling [26,39,57]. This sustained hemodynamic burden promotes left ventricular hypertrophy, concentric remodeling, and interstitial fibrosis, which in turn impair myocardial relaxation and reduce ventricular compliance. The resulting increase in left ventricular filling pressure manifests as diastolic dysfunction [54]. Consistent with this mechanism, numerous studies have demonstrated significant associations between baPWV and echocardiographic parameters of left ventricular diastolic function, particularly reduced early diastolic mitral annular velocity (e′), increased E/e′ ratio, and elevated left ventricular filling pressure, independent of conventional risk factors [51,58,59,60,61,62,63]. However, because much of the available evidence is observational, these associations do not establish a direct causal relationship between increased baPWV and the development of diastolic dysfunction. Collectively, current evidence supports a consistent association between baPWV and left ventricular diastolic dysfunction and suggests that baPWV may provide complementary information regarding subclinical cardiac structural and functional abnormalities, although its role in the early detection of diastolic dysfunction requires further prospective validation.

### 7.4. Chronic Kidney Disease

Chronic kidney disease (CKD) is a major manifestation of hypertension-mediated target organ damage and is closely associated with increased arterial stiffness. Elevated baPWV reflects structural and functional changes in the arterial wall that contribute to increased pulsatile pressure transmission to the renal microcirculation. Because the kidneys are characterized by a low-resistance vascular bed, excessive pulsatile hemodynamic stress can damage glomerular capillaries, leading to progressive renal injury [64,65]. In addition, arterial stiffening increases systolic blood pressure and pulse pressure while reducing diastolic perfusion pressure, thereby impairing renal microvascular perfusion and accelerating nephron loss [65,66]. Numerous cross-sectional studies have demonstrated significant associations between elevated baPWV and reduced estimated glomerular filtration rate (eGFR), increased urinary albumin excretion, and the presence of CKD [67,68,69]. These associations have been consistently observed in community-based populations as well as in patients with hypertension, diabetes mellitus, and established CKD. Prospective cohort studies have further demonstrated that increased baPWV is associated with accelerated decline in renal function and a higher risk of incident CKD independent of traditional risk factors [33,70,71,72]. However, these observational findings do not establish that increased arterial stiffness directly causes renal injury or CKD progression, and the relationship is likely bidirectional, as impaired renal function itself may promote arterial stiffening.

### 7.5. Cerebrovascular Disease

Cerebrovascular disease is a major manifestation of hypertension-mediated target organ damage and is closely associated with increased arterial stiffness. Elevated baPWV reflects structural and functional alterations of the arterial wall that increase central pulse pressure and the transmission of excessive pulsatile energy into the cerebral circulation. Because the cerebral microvasculature is a high-flow, low-resistance vascular bed, excessive pulsatile stress can damage small cerebral vessels, resulting in endothelial dysfunction, impaired autoregulation, blood–brain barrier disruption, and progressive cerebral small vessel disease [64,73]. Numerous cross-sectional studies have demonstrated significant associations between elevated baPWV and imaging markers of cerebrovascular damage, including silent cerebral infarction, white matter hyperintensities, enlarged perivascular spaces, cerebral microbleeds, and lacunar infarction [74,75,76,77,78]. Furthermore, higher baPWV has been consistently associated with a greater burden of cerebral small vessel disease on brain magnetic resonance imaging, including white matter hyperintensities, silent lacunar infarctions, cerebral microbleeds, and enlarged perivascular spaces [76,79]. However, these findings are predominantly observational and do not establish a direct causal relationship between increased arterial stiffness and cerebral microvascular injury. Rather, baPWV may serve as a vascular marker associated with the presence and burden of subclinical cerebrovascular damage.

### 7.6. Cognitive Impairment

**Figure 1:**
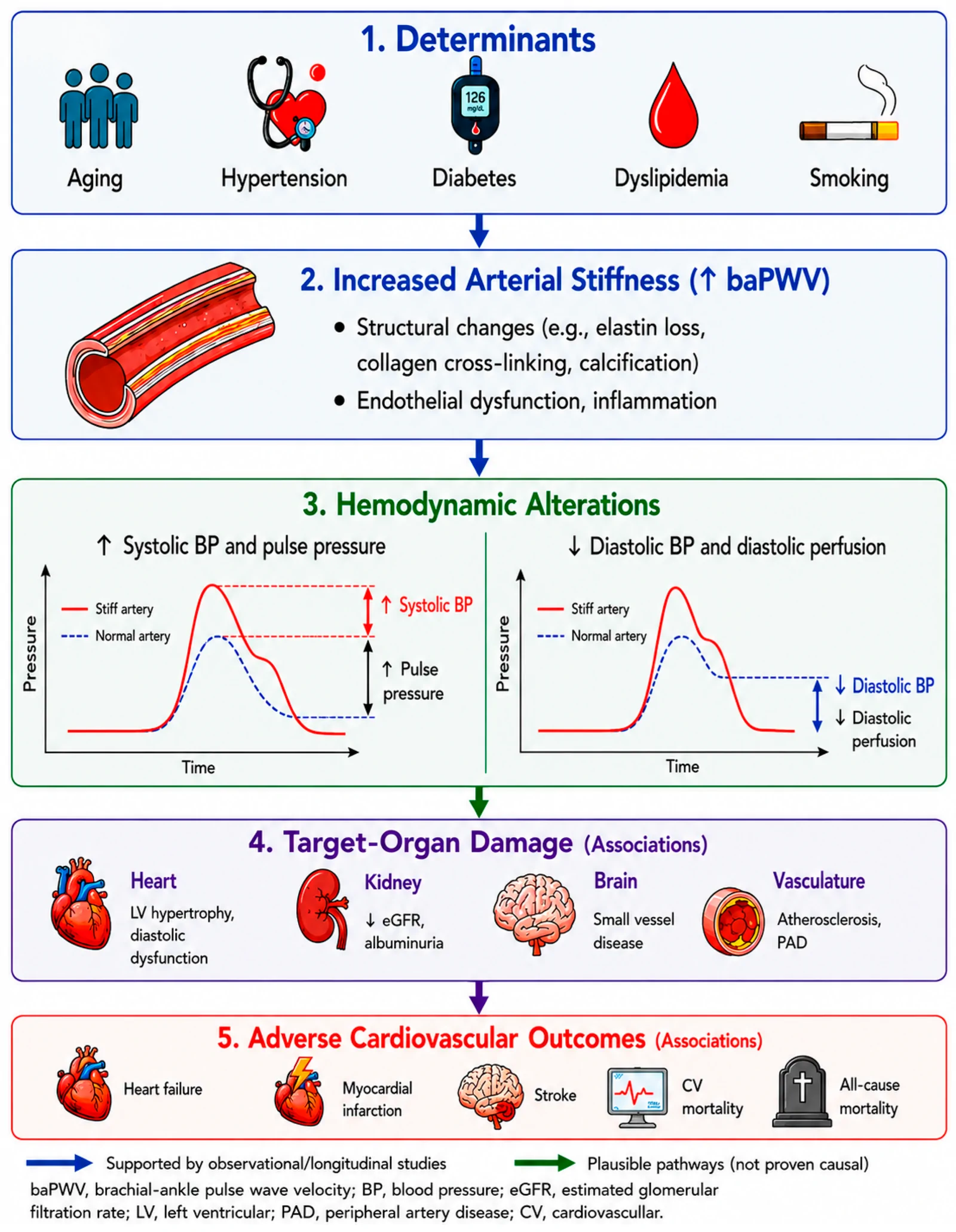
Pathophysiological framework linking arterial stiffness to target-organ damage and adverse cardiovascular outcomes. Aging and cardiovascular risk factors are associated with structural and functional vascular alterations, including elastin fragmentation, collagen cross-linking, advanced glycation end-product accumulation, vascular calcification, chronic inflammation, and endothelial dysfunction, which are associated with increased arterial stiffness. Increased arterial stiffness may alter pulsatile hemodynamics and thereby provide plausible mechanistic links to cardiac, renal, cerebral, and coronary abnormalities. Observational and longitudinal studies support associations between increased arterial stiffness and baPWV and target-organ damage and adverse cardiovascular outcomes; however, several mechanistic pathways illustrated by the arrows are pathophysiologically plausible or inferential and should not be interpreted as established causal relationships. baPWV, brachial-ankle pulse wave velocity.

Cognitive impairment is increasingly recognized as an important consequence of vascular aging and hypertension-mediated target organ damage [56,80]. Increased arterial stiffness, reflected by elevated baPWV, augments central pulse pressure and facilitates the transmission of excessive pulsatile energy into the cerebral microcirculation [6]. This process contributes to endothelial dysfunction, impaired cerebrovascular autoregulation, disruption of the blood–brain barrier, and chronic cerebral hypoperfusion, ultimately promoting cerebral small vessel disease and cognitive decline [73]. Numerous cross-sectional studies have demonstrated that higher baPWV is associated with poorer global cognitive performance and deficits in multiple cognitive domains, including executive function, processing speed, attention, and memory [81,82,83,84,85]. These associations have been consistently observed in community-based populations as well as in patients with hypertension, diabetes mellitus, chronic kidney disease, and other cardiovascular risk factors. As discussed above, higher baPWV has been associated with cerebral small-vessel disease [79,86,87]. Collectively, these findings indicate that increased arterial stiffness, as assessed by baPWV, is closely associated with both subclinical brain injury and cognitive impairment. Figure 1 illustrates the pathophysiological links between arterial stiffness and cardiovascular target organ damage.

## 8. Clinical Relevance and Potential Applications of baPWV

### 8.1. Cardiovascular Risk Stratification

Accurate cardiovascular risk stratification is essential for identifying individuals who are most likely to benefit from intensive preventive strategies. Contemporary cardiovascular risk prediction models, including the Framingham Risk Score, SCORE2, SCORE2-Older Persons (SCORE2-OP), and the recently developed PREVENT equations, primarily estimate cardiovascular risk based on demographic characteristics and conventional cardiovascular risk factors, such as age, sex, blood pressure, smoking, diabetes mellitus, and lipid profiles [88,89]. Although these models provide reasonable population-level risk estimates, they do not directly assess cumulative vascular damage or biological vascular aging. Consequently, individuals with similar traditional risk factor profiles may have substantially different levels of arterial stiffness and cardiovascular risk.

baPWV has emerged as a practical marker that complements conventional cardiovascular risk assessment by directly reflecting arterial stiffness and cumulative vascular injury. Because arterial stiffening represents the integrated effects of aging, hypertension, diabetes mellitus, dyslipidemia, chronic kidney disease, smoking, inflammation, and other cardiovascular risk factors, baPWV provides information beyond individual risk factors alone [11]. Unlike conventional risk scores, which estimate future risk based on statistical models, baPWV evaluates the current biological status of the arterial tree and therefore serves as a surrogate marker of vascular age [25]. Numerous prospective cohort studies have demonstrated that elevated baPWV is independently associated with future cardiovascular events and all-cause mortality after adjustment for conventional cardiovascular risk factors [7,9,33,34,90,91,92,93,94,95,96,97,98,99]. These findings have been consistently observed in general-population cohorts [34,99], and patients with hypertension [94,95], diabetes mellitus [90,91], coronary artery disease [92,93], chronic kidney disease [33,98], and heart failure [96,97].

Several studies have shown that incorporating baPWV into conventional cardiovascular risk prediction models significantly improves risk discrimination and reclassification [100,101,102,103,104]. In addition to traditional risk scoring systems [104], baPWV provides incremental predictive value when combined with inflammatory biomarkers such as C-reactive protein [100], noninvasive imaging modalities including coronary artery computed tomography [101] and carotid ultrasonography, and functional cardiovascular assessments [102,103]. Some studies suggest that adding baPWV to established risk assessment tools may improve risk discrimination or reclassification [105]. However, statistically significant improvements in discrimination or reclassification metrics, including the C-statistic/area under the receiver-operating-characteristic curve, net reclassification improvement, and integrated discrimination improvement, do not necessarily indicate clinically meaningful improvement in risk assessment or patient management. The reported improvements have generally been modest and heterogeneous, and whether these incremental predictive gains translate into changes in clinical decision-making or improved patient outcomes remains to be established in prospective impact studies. Table 2 summarizes prognostic evidence for baPWV from major meta-analyses and representative cohort studies.

### 8.2. Hypertension Management

Hypertension and arterial stiffness are closely interrelated through a bidirectional pathophysiological relationship [39]. Chronic elevation of blood pressure accelerates structural and functional changes in the arterial wall, including elastin fragmentation, collagen deposition, vascular calcification, and endothelial dysfunction, leading to increased arterial stiffness [39]. Conversely, increased arterial stiffness augments central systolic blood pressure and pulse pressure by accelerating pulse wave propagation and early wave reflection, thereby further increasing left ventricular afterload and perpetuating hypertension [105]. This vicious cycle contributes to the development of hypertension-mediated target organ damage and substantially increases cardiovascular risk. baPWV provides a simple and reliable assessment of arterial stiffness and therefore offers clinically relevant information beyond conventional blood pressure measurements. Patients with similar office or ambulatory blood pressure levels may exhibit markedly different degrees of arterial stiffness and vascular aging, resulting in substantial differences in cardiovascular risk [35]. Because arterial stiffness reflects cumulative vascular injury rather than blood pressure alone, baPWV may complement conventional blood pressure measurements in evaluating the overall vascular effects of antihypertensive therapy [26].

Several studies have demonstrated that effective blood pressure control through lifestyle modification and antihypertensive treatment is accompanied by a reduction in baPWV, indicating improvement in arterial stiffness [110,111,112,113,114]. The magnitude of baPWV reduction generally parallels improvements in blood pressure, although certain antihypertensive agents, particularly those targeting the renin–angiotensin system, may exert additional beneficial effects on arterial stiffness beyond blood pressure lowering [112,113,114]. Changes in baPWV during antihypertensive or lifestyle interventions may provide information on vascular responses to treatment [26]. However, these associations should not be interpreted as evidence that targeting baPWV reduction itself improves cardiovascular outcomes, and prospective trials of baPWV-guided treatment strategies are needed. Although prospective evidence demonstrating that baPWV-guided treatment improves clinical outcomes remains limited, its simplicity, reproducibility, and noninvasive nature support its potential clinical utility as a complementary vascular marker, rather than an established treatment-guiding tool, in hypertension management.

### 8.3. Serial Measurement and Therapeutic Response Monitoring

Although a single assessment of arterial stiffness provides important prognostic information, serial evaluation may offer complementary information by tracking temporal changes in vascular function and structure [115]. Arterial stiffness is a dynamic vascular biomarker that is influenced by aging, blood pressure, lifestyle factors, pharmacological treatment, and the progression of cardiovascular and metabolic disorders. Consequently, repeated assessment of arterial stiffness indices may better characterize vascular aging and treatment-induced changes than a single measurement, thereby providing additional information for cardiovascular risk assessment and longitudinal monitoring of vascular health [115]. Several longitudinal studies have demonstrated that persistently elevated baPWV or an increase in baPWV during follow-up is associated with a significantly higher risk of future cardiovascular events, heart failure, stroke, chronic kidney disease progression, and all-cause mortality compared with stable or reduced baPWV [92,108]. Conversely, improvement in baPWV following intensive blood pressure control [116], weight reduction [117], regular exercise [118], smoking cessation [119], or treatment with statin [120] has been associated with favorable changes in vascular function and a lower cardiovascular risk profile. Because baPWV measurement is simple, noninvasive, inexpensive, and highly reproducible, serial evaluation is feasible in routine clinical practice and health screening programs [6]. Monitoring temporal changes in baPWV may therefore provide complementary information beyond conventional risk factor assessment and may help characterize vascular responses to therapeutic interventions and longitudinal changes in vascular health [30,121]. However, changes in baPWV should be interpreted cautiously because baPWV is influenced by concurrent blood pressure and short-term hemodynamic conditions. Therefore, a reduction in baPWV does not necessarily indicate structural reversal of arterial stiffness and should be interpreted together with changes in blood pressure and potential measurement variability.

### 8.4. baPWV Variability

Visit-to-visit variability in baPWV has recently emerged as a potential marker of dynamic vascular changes. baPWV variability can be quantified using measures such as the standard deviation, coefficient of variation, or variability independent of the mean across repeated measurements. Preliminary prospective evidence suggests that greater visit-to-visit baPWV variability may be associated with an increased risk of cardiovascular events beyond baseline baPWV [109]. However, current evidence is limited and requires independent validation. Moreover, the optimal frequency and spacing of assessments, the minimum number of repeated measurements required to reliably estimate baPWV variability, reproducibility requirements, and the magnitude of variability that should be considered clinically actionable remain undefined.

### 8.5. Combined Use of Ankle-Brachial Index

**Figure 2:**
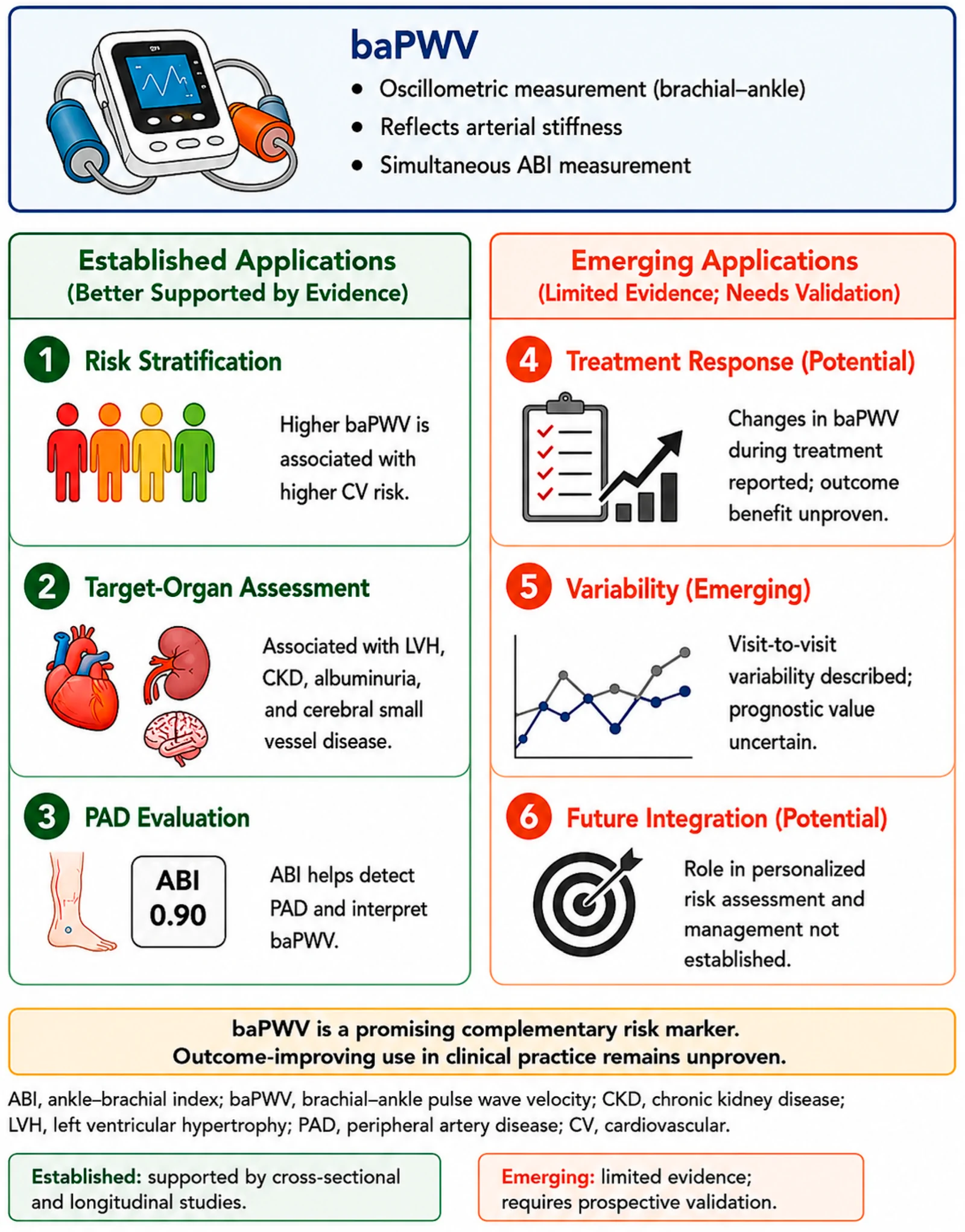
Current and emerging clinical applications of baPWV. baPWV is a simple and reproducible measure of arterial stiffness that is associated with cardiovascular risk factors, target-organ damage, and adverse cardiovascular outcomes. A relatively established body of observational and longitudinal evidence supports the prognostic value of baPWV and its potential complementary role in cardiovascular risk stratification and vascular health assessment. Simultaneous measurement of the ankle-brachial index provides additional information regarding peripheral artery disease and aids interpretation of baPWV. In contrast, serial baPWV measurements for treatment-response monitoring and visit-to-visit variability as a prognostic marker are emerging areas of investigation, with limited evidence and a need for further prospective validation. Similarly, baPWV-guided treatment and personalized cardiovascular prevention have not been demonstrated to improve clinical outcomes and should be considered potential future applications rather than established clinical uses. baPWV, brachial-ankle pulse wave velocity.

An important practical advantage of baPWV measurement is that it simultaneously provides the ankle-brachial index (ABI) without requiring additional equipment or examination time [25]. ABI is a well-established, simple, and noninvasive marker for the diagnosis of peripheral artery disease (PAD), with an ABI value of <0.90 indicating significant lower-extremity arterial obstruction [122]. Because PAD is a manifestation of systemic atherosclerosis, a reduced ABI is strongly associated with an increased risk of myocardial infarction, stroke, cardiovascular mortality, and all-cause mortality [123,124]. Therefore, the ability to obtain both baPWV and ABI in a single examination allows clinicians to evaluate two complementary aspects of vascular health: arterial stiffness, which reflects functional and structural vascular aging, and lower-extremity arterial obstruction, which reflects advanced atherosclerotic disease. This combined assessment provides a more comprehensive evaluation of vascular status than either parameter alone [125,126,127]. Furthermore, measuring ABI concurrently with baPWV is particularly valuable because severe PAD may falsely lower baPWV values owing to reduced pulse wave transmission distal to significant arterial stenosis. In such cases, ABI helps identify patients in whom baPWV should be interpreted with caution [25,30]. Consequently, the simultaneous assessment of baPWV and ABI may provide a practical and complementary approach to cardiovascular risk assessment by integrating information on both arterial stiffness and peripheral atherosclerotic burden. The clinical applications of baPWV are summarized in Figure 2.

## 9. Future Perspectives

Although substantial evidence supports the role of baPWV as a marker of arterial stiffness and cardiovascular risk, several important questions remain to be addressed. Future research should focus on establishing standardized measurement protocols, clinically meaningful cutoff values, and optimal assessment intervals across different populations. Further prospective studies are needed to independently validate the prognostic significance of baPWV variability and to determine whether it provides incremental information beyond mean or baseline baPWV. Standardization of variability metrics and establishment of the minimum number and optimal spacing of repeated measurements will also be necessary before baPWV variability can be considered for clinical application. Another important area of investigation is whether baPWV-guided therapeutic strategies can improve clinical outcomes by facilitating individualized risk assessment and treatment optimization. Advances in artificial intelligence and digital health technologies may further enhance the potential clinical applicability of baPWV by enabling automated interpretation, longitudinal trend analysis, and integration with electronic health records and other cardiovascular biomarkers. Combining baPWV with complementary vascular assessments, such as the ABI, vascular imaging, circulating biomarkers, and genetic or omics-based risk profiling, may provide a more comprehensive evaluation of vascular health and cardiovascular risk. With further validation in prospective studies and clinical impact trials, baPWV may have the potential to contribute to personalized cardiovascular risk assessment and longitudinal vascular monitoring.

The generalizability of the current evidence also warrants careful consideration. Much of the epidemiological and prognostic evidence for baPWV has been derived from East Asian populations, particularly Japan, China, and Korea, where automated oscillometric baPWV devices are widely used. Although studies in other ethnic and geographic populations have generally supported the association between arterial stiffness and cardiovascular risk, validation of baPWV itself outside East Asia remains comparatively limited. Moreover, absolute baPWV values may be influenced by age, blood pressure, body size, ethnicity, measurement methodology, and device-specific algorithms. Therefore, normative distributions and proposed prognostic thresholds derived from East Asian cohorts should not be assumed to be directly transferable to other populations or healthcare settings. Cross-device comparability also requires consideration because differences in path-length estimation, waveform acquisition, and signal-processing algorithms may affect absolute baPWV values. Further prospective studies using standardized measurement protocols across ethnically and geographically diverse populations are needed to establish population-specific reference ranges, evaluate cross-device comparability, and determine whether currently proposed prognostic thresholds can be generalized beyond the populations in which they were originally derived.

Despite accumulating evidence supporting the prognostic value of baPWV, its current position in major international clinical guidelines remains limited. Carotid-femoral PWV (cfPWV) continues to be regarded as the reference-standard noninvasive measure of arterial stiffness and is the PWV method most commonly addressed in Western hypertension and cardiovascular prevention guidelines. In contrast, baPWV has been more extensively studied and clinically adopted in East Asian countries but has not yet been routinely incorporated into major Western cardiovascular risk algorithms or guideline-directed treatment strategies. Thus, at present, baPWV should be considered a practical complementary marker of vascular stiffness and cardiovascular risk rather than a replacement for established risk assessment tools or a stand-alone measure for guiding treatment decisions. Although baPWV has demonstrated prognostic value and may provide incremental predictive information beyond conventional risk factors in selected populations, these findings should not be equated with demonstrated clinical benefit. Whether baPWV-guided risk stratification or treatment decisions improve cardiovascular outcomes remains to be established in prospective clinical impact studies.

## 10. Conclusions

baPWV is a simple, noninvasive, and reproducible measure of arterial stiffness with substantial evidence supporting its association with target-organ damage and adverse cardiovascular outcomes. Its ease of measurement and simultaneous assessment of blood pressure and ABI provide practical advantages, particularly in large-scale screening and longitudinal vascular assessment. However, cfPWV remains the reference-standard measure of arterial stiffness in major international guidelines, and baPWV has not yet been routinely incorporated into many Western cardiovascular risk algorithms or treatment strategies. Therefore, baPWV should currently be regarded as a promising complementary marker for cardiovascular risk assessment rather than an established tool for guiding treatment. Further prospective studies and clinical impact trials across diverse populations are needed to determine whether baPWV-guided risk assessment or management can improve clinical decision-making and cardiovascular outcomes.

## Figures and Tables

**Table 1:** Comparison of cfPWV and baPWV.

Parameter	cfPWV	baPWV
Measurement principle	Transit time of the pulse wave between carotid and femoral arteries	Transit time of the pulse wave between brachial arteries and ankles using oscillometric cuffs
Vascular territory	Predominantly central elastic arteries, particularly the aorta	Both central elastic arteries and peripheral muscular arteries
Physiologic relevance	More directly reflects central aortic stiffness and has a closer physiological relationship with left ventricular afterload and central hemodynamics	Reflects composite stiffness of both central and peripheral arteries; therefore, less specific to central aortic stiffness
Technique	Manual probe placement; requires a trained operator; inguinal exposure	Fully automated oscillometric cuffs; requires minimal operator training
BP dependency	Relatively dependent on BP at measurement	Dependent on BP at measurement
Reproducibility	Moderate to high; relatively operator-dependent	High; minimal operator dependency
Prognostic evidence	Reference standard; extensive meta-analytic evidence	Extensive prognostic evidence, particularly in East Asian populations
Representative clinical cutoff	>10 m/s (ESC threshold for increased arterial stiffness)	≥1800 cm/s (proposed high-risk threshold, particularly in East Asian populations)
Strengths	Gold-standard measure of arterial stiffness; directly assesses central elastic arterial stiffness; strong independent predictor of cardiovascular outcomes	Simple, automated, highly reproducible, and widely available; suitable for large-scale screening; provides simultaneous ABI measurement
Limitations	Technically demanding, requires trained operator, less suitable for large-scale screening	Includes both elastic and muscular arterial segments and is therefore less specific to central aortic stiffness; BP-dependent; may be affected by significant peripheral arterial stenosis and arterial calcification

cfPWV, carotid-femoral pulse wave velocity; baPWV, brachial-ankle pulse wave velocity; ABI, ankle-brachial index; BP, blood pressure; ESC, European Society of Cardiology.

**Table 2:** Representative studies evaluating the prognostic value of baPWV.

Study	Population/Clinical Setting	N	Follow-Up	Endpoint	Main Adjusted Effect Estimate	Incremental Predictive Value	Principal Limitations
Turin et al., 2010 [106]	General Japanese population; Takashima Study	2642	6.5 years	All-cause mortality	Highest vs. lowest baPWV tertile: HR 6.8 (95% CI, 1.4–32.8)	NR	Small number of deaths; wide CI; single Japanese population
Lee et al., 2015 [102]	Patients with suspected CAD undergoing myocardial SPECT	350	Median 441 days	CV death, ACS, or ischemic stroke	baPWV ≥ 1790 cm/s: HR 2.03 (95% CI, 1.08–6.38)	Global χ^2^ increased from 24.08 to 27.42 after adding baPWV to conventional risk factors and SPECT (*p* < 0.001)	Retrospective, single-center study; small number of events; relatively short follow-up
Ohkuma et al., 2017 [9]	Japanese participants without previous CVD; IPD meta-analysis of prospective studies	14,673	Mean 6.4 years	Incident CVD	Per 1-SD increase: HR 1.19 (95% CI, 1.10–1.29); highest vs. lowest quintile: HR 3.50 (95% CI, 2.14–5.74)	C-statistic 0.8026→0.8131 (*p* < 0.001); category-free NRI 0.247 (*p* < 0.001); IDI 0.0068 (*p* < 0.001)	Restricted to Japanese populations; observational cohorts
Lu et al., 2018 [107]	Community-based Chinese population	4251	Median 4.4 years	Fatal/nonfatal CV events and stroke	Per 1-SD increase: CV events HR 1.50 (95% CI, 1.26–1.78); stroke HR 1.53 (95% CI, 1.25–1.89)	Optimized cutoff (16.7 m/s): IDI +1.27% beyond MAP; NRI ≥ 42.2%; continuous baPWV did not significantly improve prediction beyond MAP	Relatively few events; Chinese population; data-driven cutoff
Yang et al., 2023 [99]	Chinese adults free of ASCVD, atrial fibrillation, and cancer; Kailuan cohort	47,659	Median ~3.3 years	Incident ASCVD events; all-cause mortality	HR increased progressively across baPWV categories (adjusted for conventional risk factors)	baPWV AUC/C-index significantly superior to systolic and diastolic BP for both ASCVD and all-cause mortality	Relatively short follow-up (~3 years); occupational cohort (mainly coal-industry workers) limits generalizability
Nakamura et al., 2021 [108]	Patients with CAD and impaired vascular function receiving optimal medical therapy	323	Median 35 months	Cardiac death, nonfatal MI, unstable angina, or ischemic stroke	Improvement in both FMD and baPWV was independently associated with the lowest risk of CV events	Improvement in FMD and baPWV significantly improved NRI and IDI beyond achievement of BP, LDL-C, and HbA1c targets	Small cohort; serial biomarker study; does not establish benefit of baPWV-guided therapy
Kim H.L. et al., 2026 [109]	Korean adults with repeated baPWV measurements (baseline, 1, 6, 12 mo)	794	Median 6.31 years	MACE	Visit-to-visit baPWV coefficient of variation, highest vs. lowest tertile: HR 2.38 (95% CI, 1.18–4.79)	Not formally tested (variability metric, not single-timepoint discrimination)	Single-center; requires ≥3 repeated measurements, limiting feasibility for routine practice; modest event number
Sang et al., 2021 [7]	Patients with established ASCVD; systematic review and meta-analysis of 15 cohort studies	15 studies	Variable	CV events, CV mortality, all-cause mortality	High baPWV: CV events HR 2.55 (95% CI, 1.61–4.03); CV mortality HR 2.66 (1.88–3.76); all-cause mortality HR 1.77 (1.09–2.87); per 1-SD increase for CV events HR 1.41 (1.24–1.60)	Not consistently reported across included studies	Substantial heterogeneity; variable baPWV cutoffs and endpoint definitions; predominantly East Asian evidence
Kim et al., 2022 [104]	Korean adults aged 40–79 years without established CVD; author’s own group	6359	Median 4.0 years	Cardiac death, nonfatal MI, coronary revascularization, or ischemic stroke	Higher baPWV independently associated with CV events	AUC: baPWV 0.70 vs. ACC/AHA PCE risk score 0.62 (*p* < 0.001); combination provided additional prognostic information	Retrospective, single-center design; relatively low event rate (2.0%); PCEs not race-specific for Koreans

baPWV, brachial-ankle pulse wave velocity; CAD, coronary artery disease; SPECT, single-photon emission computed tomography; CV, cardiovascular; ACS, acute coronary syndrome; CVD, cardiovascular disease; IPD, individual participant data; SD, standard deviation; MAP, mean arterial pressure; ASCVD, atherosclerotic cardiovascular disease; BP, blood pressure; AUC, area under the receiver-operating-characteristic curve; FMD, flow-mediated dilation; MI, myocardial infarction; LDL-C, low-density lipoprotein cholesterol; NR, not reported; NRI, net reclassification improvement; IDI, integrated discrimination improvement; PCE, pooled cohort equation; HR, hazard ratio; CI, confidence interval.

## Data Availability

No new data were created or analyzed in this study. Data sharing is not applicable to this article.
